# Silver- and Gold-Ordered Structures on Single-Crystal Silicon Surface After Thermal Deposition

**DOI:** 10.1186/s11671-016-1291-2

**Published:** 2016-02-04

**Authors:** Vladimir Karbivskyy, Love Karbivska, Viktor Artemyuk

**Affiliations:** G.V. Kurdymov Institute for Metal Physics of the NAS of Ukraine, blvd. Vernadsky, 36, Kiev, 03680 Ukraine

**Keywords:** Monolayer metal structures, Tunneling scanning microscopy, Silver, Gold, Thermal spraying

## Abstract

The formation mechanisms of Ag- and Au-ordered structures on single-crystal silicon (Si) (111) and Si (110) surfaces were researched using high-resolution scanning tunneling microscopy method. It was shown that different patterns of self-assembled nanostructures with very precise and regular geometric shapes can be produced by controlling process parameters of thermal metal spraying on the substrate. The surfaces of nanorelieves at each stage of deposition were researched, and the main stages of morphological transformation were fixed.

Self-ordered hexagonal pyramid-shaped nanostructures were formed at thermal deposition of gold on the Si (111), whereas only monolayer hexagonal formation could be observed on the plane Si (110). Gold monolayer flake nanostructures were obtained under certain technological parameters.

Atomically smooth Ag film cannot be obtained on the Si (111) surface by means of thermal spraying at room temperature. The formation of two-dimensional (2D) clusters takes place; heating of these clusters at several hundred degrees Celsius leads to their transformation into atomically smooth covering.

The weak interaction between Ag multilayer coatings and substrate was established that allows to clear crystal surface from metal with reproduction of the reconstructed Si (111) 7 × 7 surface by slight warming. The offered method can be used for single-crystal surface protection from destruction.

## Background

The production of nanostructures of metals on atomically clean surfaces of semiconductor single crystals is one of the main research areas for scientists who study the surface of materials and processes of thin film growth. One of the key reasons for this is that the boundary between a metal and a semiconductor is the working component of many electronic devices. However, despite many years of intensive research in this direction, the fundamental study in this area still attracts a great attention due to a number of interesting effects primarily related to obtaining technology and size effects.

It is well known that the growth mechanisms of thin metal films on various substrates are described by three basic types of growth—two-dimensional (2D) method or layer-by-layer growth (Frank–Van der Merve growth), layer-by-layer growth with further 3D islands growth (Stranski–Krastanov growth), and 3D islands growth (Volmer–Weber growth) [1]. Those three growth types may differ by varying the thermodynamic parameters [2]. For a long time, it was assumed that the metal growth on semiconductor surfaces obeys the aforementioned growth modes and is the heteroepitaxial. However, in 1996, in the study of the silver growth onto the substrate surface of GaAs (110), a new approach based on the critical film thickness was proposed [3]. It has been shown that when the film is thinner than the critical level, the shape is irregular and if thicker, it grows smooth and uniformly. Smith et al. [3] applied the two-step method that was proposed earlier by Evans et al. [4] for the silver growth on the GaAs (110) at low temperatures (~135 K) and for further annealing at room temperature. It has been found out that when the system is warmed to room temperature, initially irregular Ag film consisting of nanosized 3D clusters organizes itself in an atomically flat film. Moreover, such transition from cluster organization to atomically smooth surface is observed only when the total number of layers is at least six monolayers (ML) [3].

The physical nature of this new form of critical thickness of growth has led to a model of “electronic growing” [5]. In thin films, electrons are quantized along the surface normal. Several of the electrons can be dissipated by the film-substrate interface. Consequently, there occurs a dependence of electron energy variation on the thickness, which comprises an electrostatic potential owing to the occurrence of the electric double layer with scattered electrons at the interface and the electrons oscillation along the direction of normal to the surface. The balance between these two phenomena determines the thickness at which the total electron energy has a local minimum [5]. Thus, the film tends to flatten out and acquires the atomically flat morphology.

From this point of view, the research of formation processes of silver and gold nanostructures after thermal deposition on the surface of single-crystal semiconductor is relevant not only in the fundamental aspect, since it allows to obtain information about the principles of organizing metallic nanostructures, in particular, single-component metal structures, but also in the practical aspect. Nowadays, the noble metal nanostructures are used in many consumer technologies that exploit their physical properties, in particular, optical, conductive, antibacterial, etc. [6–10].

In this paper, we have researched the morphological peculiarities of Ag and Au nanorelief formation on the surface of single-crystal Si (111) and Si (110) planes by thermal evaporation of the metal in a vacuum.

## Methods

**Fig. 1:**
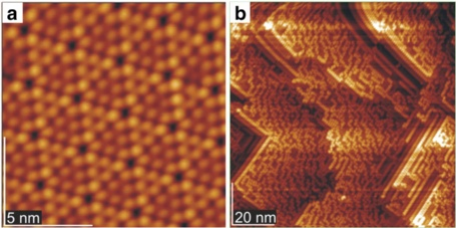
STM images of single-crystal Si surface **a** Si (111) and **b** Si (110)

Researches of the surfaces nanorelief was carried out using tunneling microscope JSPM-4610 (Japan). The operating vacuum during the experiment was not worse than 10^−8^ Pa. Single-silicon crystal wafers of Si (111) and Si (110) of 7 × 1 × 0.3 mm^3^ size were used. Preparation of single-crystal surfaces was carried out by standard methods. Initially, current with value ~0.2 A ran through the silicon plate for 24 h. In this case, the sample was heated up to ~250 °C. After that, the current through the sample was increased to 3.0 A and was held for 30 s which corresponds to the sample temperature of about 950 °C. During annealing, the temperature was monitored by an optical pyrometer. Tunnel images of the crystal surfaces (111) and (110) were obtained after cooling down (Fig. 1). All investigations in the tunneling microscope were performed at constant current mode.

The deposition of Ag and Au on the prepared surface was performed by the thermal spraying method. An atomizer represented a tungsten spiral cell with metal portion, which was in the middle of the metal cylinder with a 3-mm hole. The distance from the atomizer to the sample was about 7 cm. During the deposition, the current that ran through the tungsten spiral was ~5.0 A, which corresponds to the temperature of about 100 °C higher than the melting point of metal. The temperature of the sample was controlled by calibrated load curves. The deposition time was from few seconds to few minutes. Metal deposition on the surface of the single-crystal surface was held without heating and cooling the sample. Investigations were carried out at room temperature and the liquid nitrogen temperature.

## Results and Discussion

**Fig. 2:**
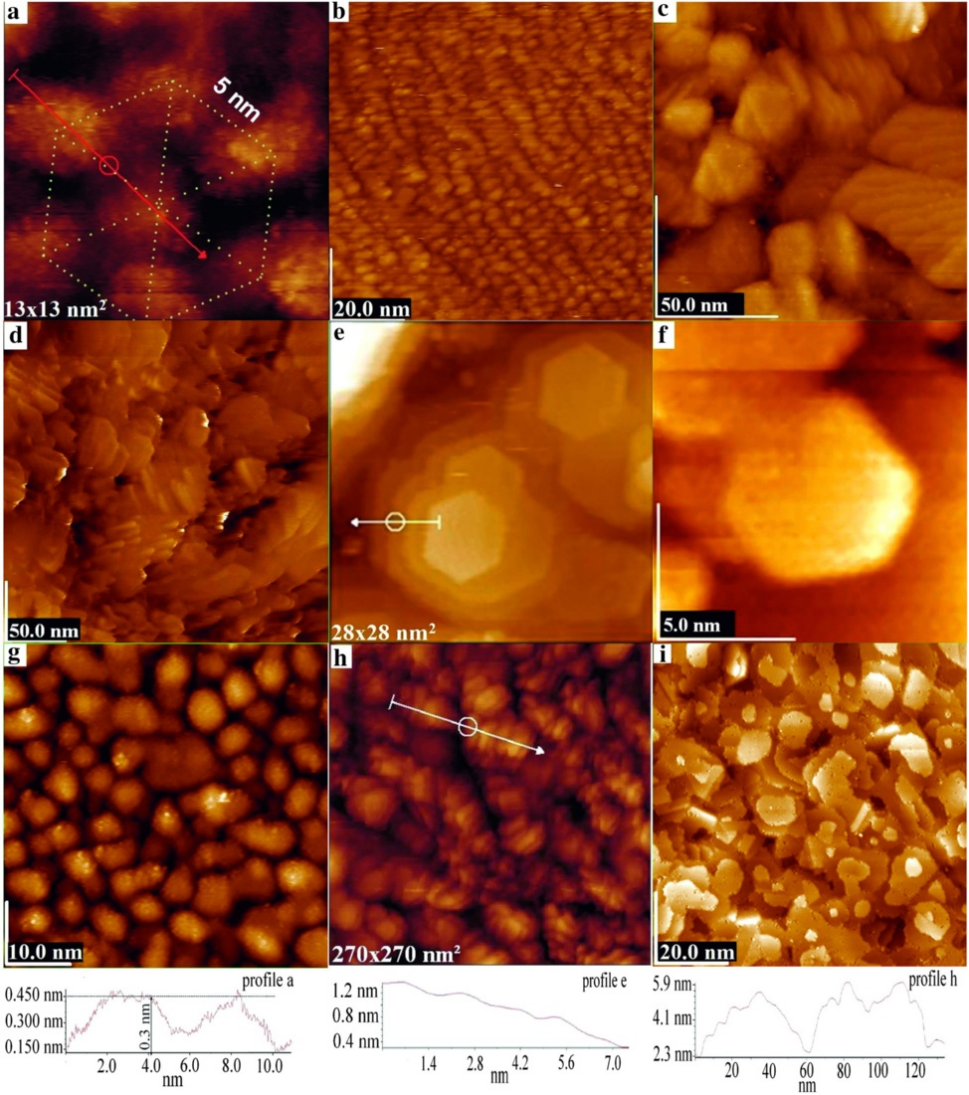
Gold nanostructures on Si (111) (**a**–**e**) and Si (110) (**f**–**i**)

The variation of the deposition parameters, namely the deposition time, the distance from the cell to the sample, the pressure in the chamber, and the temperature of melting in the cell allows stable obtaining various noble metal nanostructures shown in Fig. 2 [11].

It is possible to get a hexagonal ordering of clusters with a monomodal size distribution of ~4 nm and the hexagon side of ~5 nm (Fig. 2a). The form of such clusters is close to spherical. The ordering of this type is close to the geometry of the single-crystal surface of Si (111) 7 × 7 (Fig. 1). Figure 2b shows the ordering of chain-like clusters of about the same size with a slightly modified oval shape. Under certain technological conditions, we have obtained leaf-like gold nanostructures (Fig. 2c). The observed pattern of formation and growth of such structures has a fractal nature. A considerable part of the surface showed the cluster conservatism. Prolonged deposition time (about 1 min) led to needle-like crystals growth (Fig. 2d). The average length of the needle-like crystals was about 20 nm, and the diameter was ~7 nm. There was a slight ordering of nanocrystals in the chosen direction. Surface irregularities in assessing the height difference were no more than 3 nm. An interesting fact was a steady formation of obtained hexagonal-pyramid structures (Fig. 2e) with steps of about 0.15 nm. Hexagonal pyramid formation over the entire area of the sample had different height and upper platform area due to the uneven flow of sorption particles on the crystal surface; however, their coincidence was fairly often observed (as shown in the Fig. 2e). As it has been previously shown [11], hexagonal pyramidal gold structures are stably obtained only on the Si (111) plane, while on Si (110) plane, we have obtained the structures shown in Fig. 2f, which are characterized by the hexagon without pyramid formation.

Figure 2g shows the most typical surface landscape by the thermal deposition on the single-crystal silicon surface with minor deposition times and vacuum up to 10^−6^ Pa. The cluster formation with a small spread in size in the range of 6–8 nm was observed. In this case, increasing the deposition time led to cluster conglomerate formation (Fig. 2h). Such metamorphoses were characterized by transformation of individual cluster ensembles into a single particle with a characteristic size of about 60 nm (profile h). The height difference on the surface was still within ~3 nm.

**Fig. 3:**
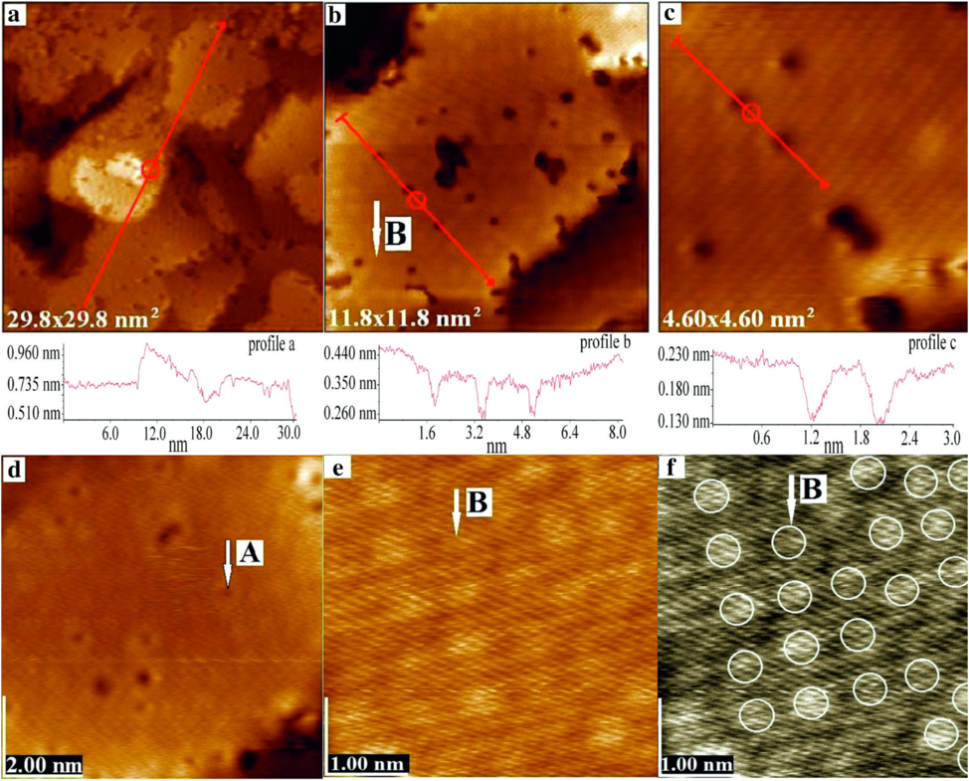
Gold nanostructures on Si (111) surface (**a**–**f**)

However, obtaining and investigation of monolayer metallic coatings at higher vacuum is of the greatest interest. As a result of methodical work, we have chosen modes of technological deposition that resulted in obtaining flaky gold nanostructures (Fig. 2i). It is clear that the linear dimensions of the flakes are in the range of 5–30 nm. A more detailed picture of these nanostructures is shown in Fig. 3. Rather high vacancy defectiveness of the flakes is observed. Detailed analysis of the flake orientation shows that many of them have no complete horizontal position, as it is shown on the profile line (Fig. 3a). The flake in the center of the pattern with the length of about 8 nm by one edge extends over ~0.16 nm above the flake plane below. This protrusion may indicate that the upper flake is formed from a single layer of atoms. The second edge of the flake superimposes on the plane, which is at the same height as the lower flake and is formed by the next flake.

In the upper left part of Fig. 3a, there is a region of the not fully formed flake. In the body of the indicated flake, there are significant areas that are not filled with atoms. Considering this and the fact that the atomic vacancies are largely observed on the edge of flakes as shown in Fig. 3b, it may be assumed that the flake self assembling is carried out by two mechanisms, i.e., atoms, deposited on the sample, through the mechanism of the thermal drift and the atom flow from the melt, which does not fully heal vacancies. And thus, as it has already been noted, the formed flakes are generally characterized by very high vacancy defectiveness.

Figure 3b shows the flake cross section, analysis of which shows a significant sag in the central flake, that resembles a dried leaf. The height difference in the middle and end points of the profile is 0.09 nm, and the flake width is about 8 nm. It is noteworthy that such a sag has been observed mostly on the horizontally oriented flakes. At the same time, the height differences between adjacent flakes, as it was noted above, were about 0.16 nm. Such a result may direct to a very important and intriguing question about the possibility of free sagging of the flakes edges. Since there is a difficulty to determine this by tunneling microscopy, it could be the subject of further studies. It should be noted that a variant of a free metal monolayer existence is contrary to the physics canons about the solid state.

Analysis of depressions (Fig. 3b) having the shape of a circle with the diameter of about 0.5 nm indicates the presence of point defects. Probably one to three gold atoms are absent in these positions. Such defect size is the most common for these samples. A detailed analysis of the vacancy depth (Fig. 3c) yields a value of about 0.11 nm, which is in proportion with the size of the atom. The electronic states in such depressions may have specific features; in particular, they may be quantized according to the quantum dots principle. Figure 3d shows that in addition to the defects with the diameter of about 0.5 nm, there are defects with the diameter of about three times less (features A, Fig. 3d), which corresponds to the size of a gold atom. Besides, there are features shown in Fig. 3b, e, and f (features B, light spots), which are associated with the peculiarities of the density of electronic states as a result of the imposition of electronic states of the upper layer and the atoms located below. For instance, such effects occur in the formation of the structure of type Si (111) 7 × 7. Due to the lack of ordering of these features, it can be concluded that the planar structures are far from crystal ordering and a faintly ordered structure of gold atoms in observable monolayers is likely to occur.

The stated information may indicate that the laying of gold atomic gas in the observed structures cannot be fully described by the mentioned schemes of nanorelief formation. Dynamic picture of atomic steady stream is equal to the difference between the flows to surface and from it. By changing the deposition technological parameters, we may get a particular morphological picture of the surface.

An entirely different situation was observed during the research of silver deposition on the surface of Si (111). The growth dynamics of silver clusters on the silicon semiconductor surface at the room temperature was studied by Kocán in [12] by the method of scanning tunneling microscopy. It has been found out that during the deposition of Ag atoms on the single-crystal silicon surface, particles quite rapidly diffuse on the surface and at first seek the places suitable for formation of the structures with increased adsorption energy. These places are edges of various protrusions, already formed islands of silver and various defects. The dynamics of the process of Ag spraying on the surface of Si (111) 7 × 7 is studied in detail in the paper [12]. Earlier researches indicate that silver atoms are randomly captured and held by halves of the unit cells that compose reconstructed surface of Si (111) 7 × 7, while the diffusion is observed within the halves of these unit cells [13, 14]. However, inter-cell jumps are very rare, that is proved by the long lifetimes of particles inside the halves of the unit cells [15]. Matsuda and Yeom [16] in the research of metastable Ag films grown on the surface of Si (111) 7 × 7 at low temperatures showed that the morphology of the film growth with a low covering degree is inconsistent with generally accepted model of electronic growing.

The paper [17] shows the influence of the deposition temperature on the growth morphology of such structures, as well as the influence of some parameters on the electronic mechanism of growth. The impossibility of producing flat Ag films at the room temperature even at a critical thickness has been shown. Despite the fact that the surface of the film is locally flat, each flat region is divided by grooves.

**Fig. 4:**
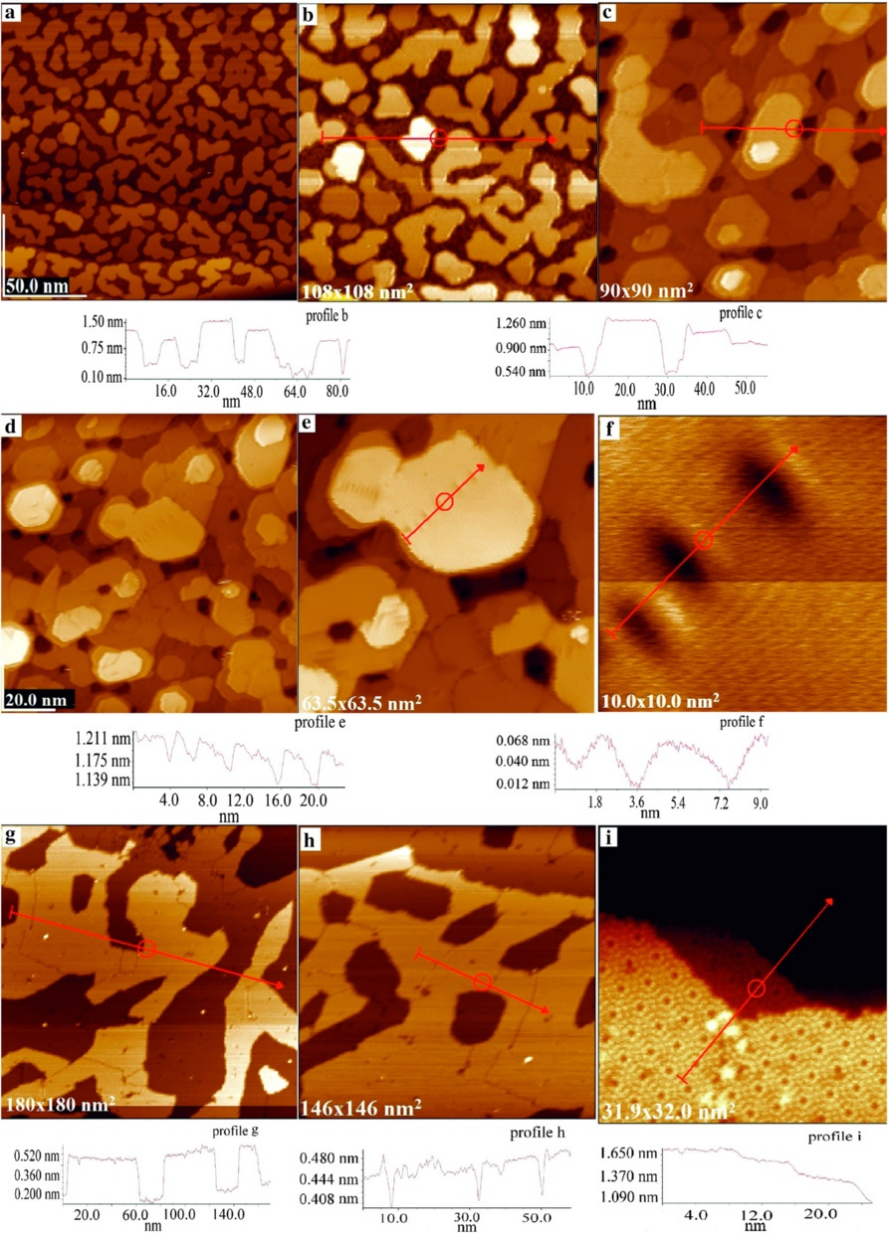
Ag nanostructures on the Si (111) (**a**–**i**)

After the first deposition at room temperature for 2 s and melt temperature of about 100° above the melting point, we obtained two-dimensional clusters on the single-crystal surface (Fig. 4a), which as seen in Fig. 4b consisted of three to four silver ML since the height difference, as seen in profile (Fig. 4b), which was about 0.7 nm. As it is evident from the Fig. 4a there are no silver clusters on the steps of the break of the planes of single-crystal silicon. Two-dimensional clusters fully cover the planes and there is no cluster that is located simultaneously on two planes of single crystal because this situation is not energetically favorable.

The surface morphology did not practically change at cooling this sample to the temperature of liquid nitrogen.

Silver deposition under the same technological conditions for 3–4 s led to the picture shown in Fig. 4c, where about six to seven metal monolayers were observed. Unlike Fig. 4a and b, in Fig. 4c, d, and f, the fusion of dimensional clusters into large clusters with clear inter-grain boundaries is observed. In order to reduce the total energy of the two-dimensional grains, the dislocation could be observed on many of them. Their detailed analysis shows that topologically, they form the depression of about 0.05 nm.

**Fig. 5:**
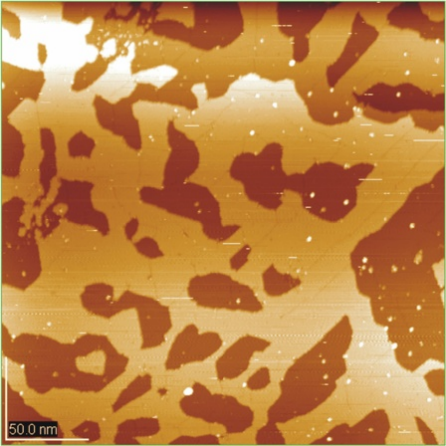
Ag nanostructures on the Si (111) (nanoart)

After short heating of such a sample for 2 min and temperature of about 350 °C, the “carpet” effect is observed. Merging of small two-dimensional clusters into large ones (Fig. 4g) took place. Further heating for 2 min at the same temperature led to the transformation into almost complete covering (Fig. 4h). Within one plane, there is a percolation effect. Sometimes, attractive structures (Fig. 5) were formed at the research of such samples, where it was possible to observe many interesting objects (pentagon, hearts, birds, etc.).

At the last research stage, we heated the sample at a temperature of about 600 °C for a few minutes. This resulted in the evaporation of deposited silver, and the reconstructed surface of single-crystal Si (111) 7 × 7 (Fig. 4i) was observed. This indicates that monolayer coverings have extremely weak chemical interaction with the surface of the single crystal, and the proposed method may be used to protect the single-crystal surfaces from destruction.

## Conclusions

The nanorelief formation mechanism of Ag and Au on the single-crystal Si surface (111) with a multi-stage thermal spraying has been researched. Surface nanorelieves at each stage of deposition and the main stages of morphological transformation have been researched and fixed. It is established that the growth of nano-gold islands on the single-crystal silicon surface is determined by the flow of substances to the surface and from it (dynamic model). Significant material stream on semiconductor substrate (without cooling it) results in complicated gold nanostructure growth mechanisms that differ from the known mechanisms. By varying the parameters of the process deposition and, as a consequence, by controlling the flow of vaporized material (Ag or Au), different pictures of self-assembled nanostructures with very precise and regular geometric shapes can be obtained.

It has been shown that during the thermal spraying of gold on the single-crystal Si surface (111), the initial stage of gold nanostructure formation has a fractal character. Further growth mechanism is characterized by nanoparticle conglomerate formation with subsequent conversion to ellipsoidal particles and further formation of needle-like nanocrystals. The next transformation stage is characterized by the crystallographic plane formation from such needle-like nanocrystals.

Self-ordered hexagonal pyramid-shaped nanostructures were formed during thermal deposition of gold on the Si (111), whereas only monolayer hexagonal formation could be observed on the plane Si (110). Gold monolayer flake nanostructures were obtained under certain technological parameters.

The gold monolayer flake nanostructures with high vacancy imperfection can be obtained under certain technological conditions.

Atomically smooth Ag film cannot be obtained on the Si (111) surface by means of thermal spraying at room temperature. The formation of 2D clusters takes place; heating of these clusters under several hundred degrees Celsius leads to their consolidation into atomically smooth covering. Obtained silver multilayer structures are satisfactorily described in the framework of an electronic-growing method.

The weak interaction between Ag multilayer coatings and substrate was established that allows to clear crystal surface from metal with reproduction of the reconstructed Si (111) 7 × 7 surface by slight warming. The offered method can be used for single-crystal surface protection from destruction.

## Abbreviations

ML: monolayer
STM: scanning tunneling microscopy
